# Ligand Binding to the FA3-FA4 Cleft Inhibits the Esterase-Like Activity of Human Serum Albumin

**DOI:** 10.1371/journal.pone.0120603

**Published:** 2015-03-19

**Authors:** Paolo Ascenzi, Loris Leboffe, Alessandra di Masi, Viviana Trezza, Gabriella Fanali, Magda Gioia, Massimo Coletta, Mauro Fasano

**Affiliations:** 1 Interdepartmental Laboratory of Electron Microscopy, Roma Tre University, Via della Vasca Navale 79, I-00146 Roma, Italy; 2 Department of Sciences, Roma Tre University, Viale Guglielmo Marconi 446, I-00146 Roma, Italy; 3 Biomedical Research Division, Department of Theoretical and Applied Sciences, University of Insubria, Via Alberto da Giussano 12, I-21052 Busto Arsizio (VA), Italy; 4 Department of Clinical Sciences and Translational Medicine, University of Roma “Tor Vergata”, Via Montpellier 1, I-00133 Roma, Italy; 5 Interuniversity Consortium for the Research on the Chemistry of Metals in Biological Systems, Via Celso Ulpiani 27, I-70126 Bari, Italy; Russian Academy of Sciences, Institute for Biological Instrumentation, RUSSIAN FEDERATION

## Abstract

The hydrolysis of 4-nitrophenyl esters of hexanoate (NphOHe) and decanoate (NphODe) by human serum albumin (HSA) at Tyr411, located at the FA3-FA4 site, has been investigated between pH 5.8 and 9.5, at 22.0°C. Values of *K*_s_, *k*_+2_, and *k*_+2_/*K*_s_ obtained at [HSA] ≥ 5×[NphOXx] and [NphOXx] ≥ 5×[HSA] (Xx is NphOHe or NphODe) match very well each other; moreover, the deacylation step turns out to be the rate limiting step in catalysis (*i*.*e*., *k*_+3_ << *k*_+2_). The pH dependence of the kinetic parameters for the hydrolysis of NphOHe and NphODe can be described by the acidic p*K*_a_-shift of a single amino acid residue, which varies from 8.9 in the free HSA to 7.6 and 7.0 in the HSA:NphOHe and HSA:NphODe complex, respectively; the p*K*>_a_-shift appears to be correlated to the length of the fatty acid tail of the substrate. The inhibition of the HSA-Tyr411-catalyzed hydrolysis of NphOHe, NphODe, and 4-nitrophenyl myristate (NphOMy) by five inhibitors (*i*.*e*., diazepam, diflunisal, ibuprofen, 3-indoxyl-sulfate, and propofol) has been investigated at pH 7.5 and 22.0°C, resulting competitive. The affinity of diazepam, diflunisal, ibuprofen, 3-indoxyl-sulfate, and propofol for HSA reflects the selectivity of the FA3-FA4 cleft. Under conditions where Tyr411 is not acylated, the molar fraction of diazepam, diflunisal, ibuprofen, and 3-indoxyl-sulfate bound to HSA is higher than 0.9 whereas the molar fraction of propofol bound to HSA is *ca*. 0.5.

## Introduction

The (pseudo-)enzymatic activity of human serum albumin (HSA) was first reported in 1951 and investigated extensively for decades. Among others, HSA displays esterase, RNA-hydrolyzing, enolase, glucuronidase, lipid peroxidase, aldolase glutathione-linked thiol peroxidase, and anti-oxidant activities. Moreover, heme binding confers to HSA globin-like (pseudo-)enzymatic properties, including detoxification of reactive nitrogen and oxygen species as well as catalase and peroxidase activities. Remarkably, HSA(-heme) (pseudo-)enzymatic properties are modulated allosterically and inhibited competitively [1,2].

Although the physiological importance of the esterase activity of HSA is obscure and the natural substrates are still unknown, HSA displays esterase activity towards several substrates including 4-nitrophenyl acetate (NphOAc), α-naphthyl acetate, phenyl acetate, 1-naphthyl *N*-methylcarbamate, β-naphthyl acetate, aspirin, ketoprofen glucuronide, carprofen acylglucuronide, cyclophosphamide, nicotinate esters, long and short-chain FA esters (*e*.*g*., 4-nitrophenyl myristate; NphOMy), octanoyl ghrelin, organophosphorus pesticides, carbaryl, 2-nitrotrifluoroacetanilide, 2-nitroacetanilide, and nerve agents [1–3].

The Lys199 and Tyr411 residues, placed in the fatty acid (FA) 7 (FA7) and 3–4 (FA3-FA4) site, respectively, are pivotal for the esterase activity of HSA; however, mechanisms for the Lys199- and Tyr411-assisted catalysis are substantially different [1–8].

At Lys199, the substrate (*e*.*g*., acetylsalicylic acid, trinitrobenzeno-sulfonates, and penicillin) is cleaved in two products; while one product is released, the other one binds covalently to the Lys199 residue [1,4,7]. Although the molecular mechanism underlying the Lys199 acetylation is unknown, it seems that its ability to attack the substrate is due to the proximity of the Lys195 residue, these two residues playing a combined and comparable chemical role. In fact, the basic form of Lys199 is likely connected to the acid form of Lys195 through a network of H-bonding water molecules with a donor-acceptor character. The presence of these water bridges is relevant for stabilizing the configuration of the FA7 site and/or promoting a potential Lys195-Lys199 proton-transfer process [6]. Since Lys199 is placed at the entrance of the FA7 site (*i*.*e*., Sudlow’s site I), ligand binding inhibits the Lys199-dependent esterase activity [3,8].

The catalytic mechanism involving the Tyr411 residue appears to be substrate-dependent. Of note, the hydrolysis of the most suitable substrate 4-nitrophenyl propionate leads to the release of both 4-nitrophenol and propionate [9]. This mechanism also applies to the hydrolysis of *N*-trans-cinnamoylimidazoles [10] and 4-nitrophenil esters of amino acids [11]. However, the Tyr411-assisted hydrolysis of NphOAc and NphOMy leads to the release of 4-nitrophenol and to Tyr411-acetylation and-myristoylation, respectively [12,13]. The strong nucleophilic nature of the phenolic oxygen of the Tyr411 residue is due to the close proximity of the Arg410 guanidine moiety that electrostatically stabilizes the reactive anionic form of the Tyr411 residue [5,14]. Since both the Arg410 and Tyr411 residues are placed in the FA3-FA4 site (*i*.*e*., Sudlow’s site II), ligand binding inhibits the HSA esterase activity [3,9,12,13]. Remarkably, the esterase activity of HSA could play a role in the inactivation of several toxins including organophosphorus compounds [3].

Present study largely extends previous investigations concerning the hydrolysis of 4-nitrophenyl esters by HSA [9,12–14]. In particular, kinetics of the HSA pseudo-enzymatic hydrolysis of 4-nitrophenyl hexanoate (NphOHe) and 4-nitrophenyl decanoate (NphODe) have been investigated between pH 5.8 and 9.5, under conditions where [HSA] ≥ 5×[NphOXx] and [NphOXx] ≥ 5×[HSA] (Xx indicates He or De). The rationale behind this selection is to investigate how the FA tail length affects the p*K*_a_ values of the ionizing group that modulates the catalysis. Furthermore, diazepam, diflunisal, ibuprofen, 3-indoxyl-sulfate, and propofol have been reported to inhibit competitively the HSA-Tyr411-catalyzed hydrolysis of NphOHe, NphODe, and 4-nitrophenyl myristate (NphOMy) (see [12] and present study). Remarkably, the molar fraction of diazepam, diflunisal, ibuprofen, and 3-indoxyl-sulfate bound to not acylated HSA is higher than 0.9 whereas the molar fraction of propofol bound to HSA is *ca*. 0.5.

## Materials and Methods

NphOHe, NphODe, NphOMy, 4-nitrophenol (NphOH), diazepam, diflunisal, ibuprofen, 3-indoxyl-sulfate, propofol, and 1,3-bis(tris(hydroxymethyl)methylamino)propane (Bis-tris propane) were obtained from Sigma-Aldrich (St. Louis, MO, USA). All chemicals were of analytical or reagent grade and were used without further purification.

HSA (from Sigma-Aldrich, St Louis, MO, USA) was essentially FA free and was used without further purification. The HSA stock solution ([HSA] = 1.2×10^-2^ M) was prepared by dissolving HSA in 2.0×10^-2^ M Bis-tris propane buffer solution (pH 7.5). The HSA concentration was determined spectrophotometrically at 279 nm (ε = 3.6×10^4^ M^–1^ cm^–1^) [1]. Then, the HSA stock solution was diluted in the Bis-tris-propane buffer (0.1 M), at the desired pH; the final pH ranged between 5.8 and 9.5. The final HSA concentration ranged between 2.0×10^-6^ M and 10×10^-4^ M.

The NphOHe, NphODe, and NphOMy solutions were prepared by dissolving the substrates in a 2.0×10^-2^ M Bis-tris propane buffer solution (pH 7.5) in the presence of 10% acetonitrile. The NphOHe, NphODe, and NphOMy concentration was determined spectrophotometrically at 400 nm (ε = 1.8×10^4^ M^–1^ cm^–l^; pH > 8.5 and 22.0°C), allowing to calculate the amount of 4-nitrophenol released from the substrate [15]. The final NphOHe, NphODe, and NphOMy concentration ranged between 2.0×10^-6^ M and 1.0×10^-4^ M. The final acetonitrile concentration was 0.5% (v/v) [9,12,13].

Kinetics and thermodynamics of the HSA-Tyr411-catalyzed hydrolysis of NphOHe, NphODe, and NphOMy were followed spectrophotometrically at 405 nm by rapid mixing the HSA solution with the NphOHe, NphODe, and NphOMy solutions [9,12,13,15].

**Fig 1:**
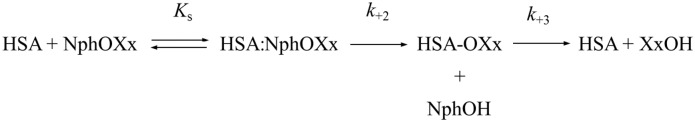
The minimum three step-mechanism for the HSA-Tyr411-catalyzed hydrolysis of NphOHe, NphODe, and NphOMy. HSA is the substrate-free protein, NphOXx is the substrate, HSA:NphOXx is the reversible protein-substrate complex, HSA-OXx is considered to be an ester formed between the acyl moiety of the substrate and the O atom of the Tyr411 phenoxyl group [14], XxOH is hexanoate or decanoate or myristate, *K*_s_ is the pre-equilibrium constant for the formation of the HSA:NphOXx complex, *k*_+2_ is the first-order acylation rate constant, and *k*_+3_ is the first-order deacylation rate constant. Xx indicates Ac or He or De or My.

Kinetics and thermodynamics of the HSA-Tyr411-catalyzed hydrolysis of NphOHe, NphODe, and NphOMy, obtained under conditions where [NphOXx] ≥ 5×[HSA] and [HSA] ≥ 5×[NphOXx], were analyzed in the framework of the minimum three step-mechanism reported in Fig. 1 [9,12–16].

Kinetics and thermodynamics of the HSA-Tyr411-catalyzed hydrolysis of NphOHe, NphODe, and NphOMy were also determined in the presence of diazepam, diflunisal, ibuprofen, 3-indoxyl-sulfate and propofol; the ligand concentration ranged between 4.0×10^-6^ M and 8.0×10^-3^ M.

Kinetic and thermodynamic parameters were obtained between pH 5.8 and 9.5, at 22.0°C.

Experiments were carried out with the SFM-200 rapid-mixing stopped-flow apparatus (BioLogic Science Instruments, Claix, France) and the Cary 50 Bio spectrophotometer (Varian Inc., Palo Alto, CA, USA).

Kinetics and thermodynamics of the HSA pseudo-esterase activity were analyzed using the GraphPad Prism program (GraphPad Software, Inc., La Jolla, CA, USA). The results are given as mean values of at least four experiments plus or minus the corresponding standard deviation.

## Results

### Pseudo-esterase activity of HSA-Tyr411

**Fig 2:**
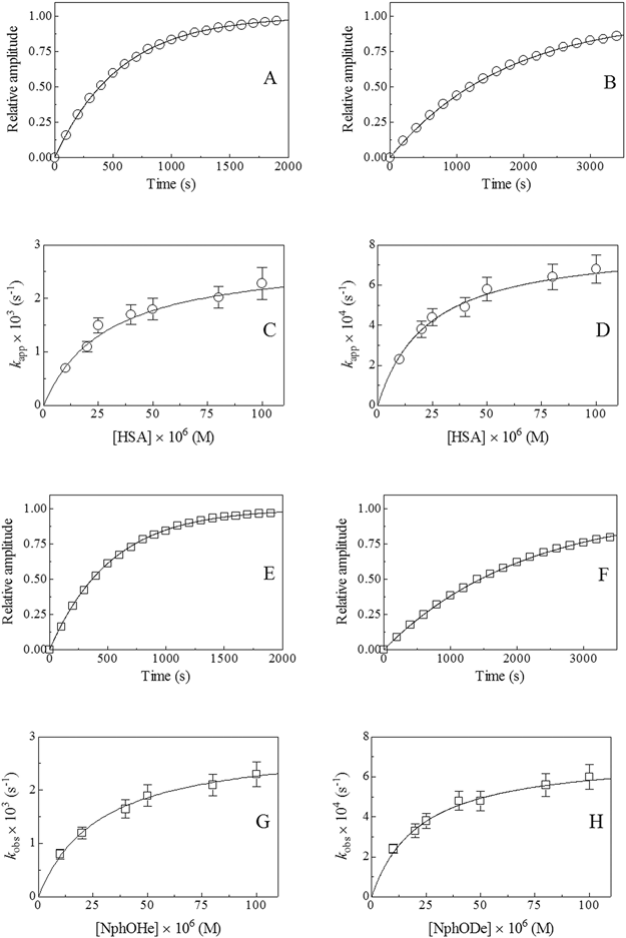
HSA-mediated hydrolysis of NphOHe and NphODe. Time course of the reaction of 2.0×10^-6^ M NphOHe (panel A) and NphODe (panel B) with 5.0×10^-5^ M HSA, *i*.*e*. [HSA] ≥ 5×[NphOHe] and [HSA] ≥ 5×[NphODe], respectively. The continuous lines were calculated according equation (1) with *k*_app_ = (1.8±0.2)×10^-3^ s^–1^ (panel A) and (5.8±0.6)×10^-4^ s^–1^ (panel B). Dependence of *k*_app_ on the HSA concentration at [HSA] ≥ 5×[NphOHe] (panel C) and [HSA] ≥ 5×[NphODe] (panel D), respectively. The continuous lines were obtained according to equation (2) with the following parameters *k*_+2_ = (2.8±0.3)×10^-3^ s^–1^ and *K*_s_ = (2.9±0.3)×10^-5^ M (panel C), and *k*_+2_ = (8.1±0.8)×10^-4^ s^–1^ and *K*_s_ = (2.3±0.3)×10^-5^ M (panel D). [NphOHe] and [NphODe] was 2.010^-6^ M and [HSA] ranged from 1.0×10^-5^ M to 1.0×10^-4^ M. Time course of the reaction of 2.0×10^-6^ M HSA with 5.0×10^-4^ M NphOHe (panel E) and NphODe (panel F)., *i*.*e*. [NphOHe] ≥ 5×[HSA] and [NphOHe] ≥ 5×[HSA], respectively. The continuous lines were calculated according to equation (6) with *k*_obs_ = (1.9±0.2)×10^-3^ s^–1^ (panel E) and (4.8±0.5)×10^-4^ s^–1^ (panel F). Dependence of *k*_obs_ on the NphOHe and NphODe concentration at [NphOHe] ≥ 5×[HSA] (panel G) and [NphODe] ≥ 5×[HSA] (panel H). The continuous lines were obtained according to equation (6) with the following parameters *k*_+2_ = (2.9±0.3)×10^-3^ s^–1^ and *K*_s_ = (2.8±0.3)×10^-5^ M (panel G), and *k*_+2_ = (7.1±0.8)×10^-4^ s^–1^ and *K*_s_ = (2.2±0.2)×10^-5^ M (panel H). The value of *k*_+3_ approximates to 0 s^–1^. [HSA] was 2.0×10^-6^ M, and [NphOHe] and [NphODe] ranged from 1.0×10^-5^ M to 1.0×10^-4^ M. Where not shown, the standard deviation is smaller than the symbol. For details, see text.

As previously reported for the HSA-Tyr411-catalyzed hydrolysis of NphOAc and NphOMy [12,13], the determination of kinetic parameters of Fig. 1 is simplified by the fact that the formation of the HSA:NphOHe and HSA:NphODe complexes can be treated as a rapid equilibrium process. Indeed, no lag phase occurs in the release of NphOH from NphOHe and NphODe in the presence of HSA (Fig. 2), indicating that the equilibration of HSA:NphOHe and HSA:NphODe with HSA and NphOHe and NphODe, respectively, is complete within 1.4 ms (*i*.*e*., the “dead-time” of the rapid-mixing stopped-flow apparatus). Moreover, the rate of NphOH release from NphOHe and NphODe catalyzed by HSA-Tyr411 is unaffected by the addition of NphOH (up to 1.0×10^-4^ M) in the reaction mixtures (data not shown), indicating that the acylation step is essentially irreversible. If NphOH had affected the HSA-Tyr411 catalyzed hydrolysis of NphOHe and NphODe, the classical product (*i*.*e*., NphOH) inhibition behavior would have been observed.

When [HSA] ≥ 5×[NphOHe] and [HSA] ≥ 5×[NphODe], the reaction of HSA with NphOHe and NphODe displays a mono-exponential time-course (Fig. 2, panels A and B). Values of the pseudo-first order rate constant for the HSA-Tyr411-catalyzed hydrolysis of NphOHe and NphODe (*i*.*e*., of NphOH release; *k*_app_) were obtained according to equation (1) [9,12,13]:

$${\left[\text{NphOXx}\right]}_{\text{t}}={\left[\text{NphOXx}\right]}_{\text{i}}\times (1-{\text{e}}^{-k_{\text{app}}\times t})$$ (1)

where Xx is De or He. Values of *k*_app_ were independent of the NphOHe and NphODe concentration under conditions where [HSA] ≥ 5×[NphOHe] and [HSA] ≥ 5×[NphODe].

Values of *K*_s_ and *k*_+2_ for the HSA-Tyr411-catalyzed hydrolysis of NphOHe and NphODe (see Table 1) were obtained from the hyperbolic plots of *k*_app_ as a function of the HSA concentration (Fig. 2, panels C and D) according to equation (2) [9,12,13]:

**Table 1:** Values of catalytic parameters for the HSA-Tyr411-catalyzed hydrolysis of NphOHe, NphODe, and NphOMy, at pH 7.5 and 22.0°C.

Substrate	[HSA] ≥ 5×[NphOXx]			[NphOXx] ≥ 5×[HSA]		
	*K*_s_ (μM)	*k*_+2_ (s^–1^)	*k*_+2_ */K*_s_ (M^–1^ s^–1^)	*K*_s_ (μM)	*k*_+2_ (s^–1^)	*k*_+2_ */K*_s_ (M^–1^ s^–1^)
NphOAc ^a^	(4.8±0.5)×10^-4^	(3.9±0.4)×10^-1^	(8.1±0.9)×10^2^	(4.7±0.5)×10^-4^	(4.2±0.4)×10^-1^	(8.4±0.9)×10^2^
NphOHe ^b^	(2.9±0.3)×10^-5^	(2.8±0.3)×10^-3^	(9.7±1.3)×10^1^	(2.8±0.3)×10^-5^	(2.9±0.3)×10^-3^	(1.0±0.2)×10^2^
NphODe ^b^	(2.3±0.2)×10^-5^	(8.1±0.8)×10^-4^	(3.5±0.5)×10^1^	(2.2±0.2)×10^-5^	(7.1±0.8)×10^-4^	(3.3±0.5)×10^1^
NphOMy ^c^	(2.6±0.3)×10^-5^	(1.6±0.2)×10^-4^	6.2±0.6	(2.5±0.3)×10^-5^	(1.5±0.2)×10^-4^	5.9±0.6

^a^ From [13].

^b^ Present study.

^c^ From [12].

$$k_{\text{app}}=\left(k_{+2}\times [\text{HSA}]\right)/\left(K_{\text{s}}+[\text{HSA}]\right)$$ (2)

When [NphOHe] ≥ 5×[HSA] and [NphODe] ≥ 5×[HSA], the reaction of NphOHe and NphODe with HSA displays a mono-exponential time course (Fig. 2, panels E and F). Values of the pseudo-first-order rate constant for the HSA-Tyr411-catalyzed hydrolysis of NphOHe and NphODe (*i*.*e*., of NphOH release; *k*_obs_) were obtained according to equation (3) [9,12,13]:

$${\left[\text{NphOXx}\right]}_{\text{t}}={\left[\text{NpHOXx}\right]}_{\text{i}}\times (1-{\text{e}}^{-k_{\text{obs}}}{}^{\times t})$$ (3)

Values of *k*_obs_ are independent of the HSA concentration when [NphOHe] ≥ 5×[HSA] and [NphODe] ≥ 5×[HSA].

When *k*_+2_ ≥ 5×*k*_+3_, the differential equations arising from Fig. 1 may be solved [12,13,16,17] to describe the time course of NphOH release at the early stages of the reaction. The resulting expression is given in eqs (4)–(6) [12,13,16,17]:

$$\left[\text{NphOH}]=(\alpha \times [\text{HSA}]\times (\text{1}-{\text{e}}^{-k_{\text{obs}}}{}^{\times t}))+(\left(k_{\text{cat}}\times [\text{HSA}]\times [\text{NphOXx}]\times t\right)/\left(K_{\text{m}}+[\text{NphOXx}]\right)\right)$$ (4)

where

$$\alpha ={\left(\left(k_{+2}\times [\text{NphOXx}]\right)/\left((k_{+2}+k_{+3})\times (K_{\text{m}}+[\text{NphOXx}])\right)\right)}^{2}$$ (5)

and

$$k_{\text{obs}}=(\left(k_{+2}\times [\text{NphOXx}]\right)/\left(K_{\text{s}}+[\text{NphOXx}]\right))+k_{+3}$$ (6)

As predicted from eqs (4)–(6), a “burst” phase of NphOH release of amplitude α with the first order rate constant *k*_obs_ occurs. Values of α, obtained at [NphOHe] ≥ 5×[HSA] and [NphODe] ≥ 5×[HSA], range between 0.99 and 1.03 (S1 Table), indicating that the HSA:NphOXx:NphOH stoichiometry is 1:1:1. Moreover, the time course of the “burst” phase of NphOH release is a first-order process for more than 95% of its course (Fig. 2, panels E and F) as estimated from residual analysis. Values of *k*_obs_ are independent of the HSA concentration when [NphOHe] ≥ 5×[HSA] and [NphODe] ≥ 5×[HSA]. Values of *K*_s_ and *k*_+2_ (see Table 1) were determined from hyperbolic plots of *k*_obs_ *versus* [NphOXx] (Fig. 2, panels G and H) according to equation (6) [16,17]. Under all the experimental conditions, the *y*-intercept of the hyperbola described by equation (6) was < 2×10^-6^ s^–1^, thus indicating that the value of *k*_+3_ is at least 100-fold smaller than that of *k*_obs_ obtained at the lowest NphOHe and NphODe concentration (*i*.*e*., *k*_+3_ < 2×10^-6^ s^–1^).

As predicted from Fig. 1, values of *K*_s_ and *k*_+2_ obtained under conditions where [HSA] ≥ 5×[NphOHe] and [HSA] ≥ 5×[NphODe] from equation (2) are in excellent agreement with those obtained under conditions where [NphOHe] ≥ 5×[HSA] and [NphODe] ≥ 5×[HSA] from equation (6) (Table 1). Moreover, data here reported indicate that the deacylation process is rate limiting in the HSA-Tyr411-catalyzed hydrolysis of NphOHe and NphODe, as previously reported for NphOAc and NphOMy [12,13] (*i*.*e*., *k*_+3_ << *k*_+2_). Furthermore, values of *K*_s_ and *k*_+2_ here obtained for the HSA-Tyr411-catalyzed hydrolysis of NphOHe and NphODe agree with those previously reported [9].

### pH effects on the pseudo-esterase activity of HSA-Tyr411

**Fig 3:**
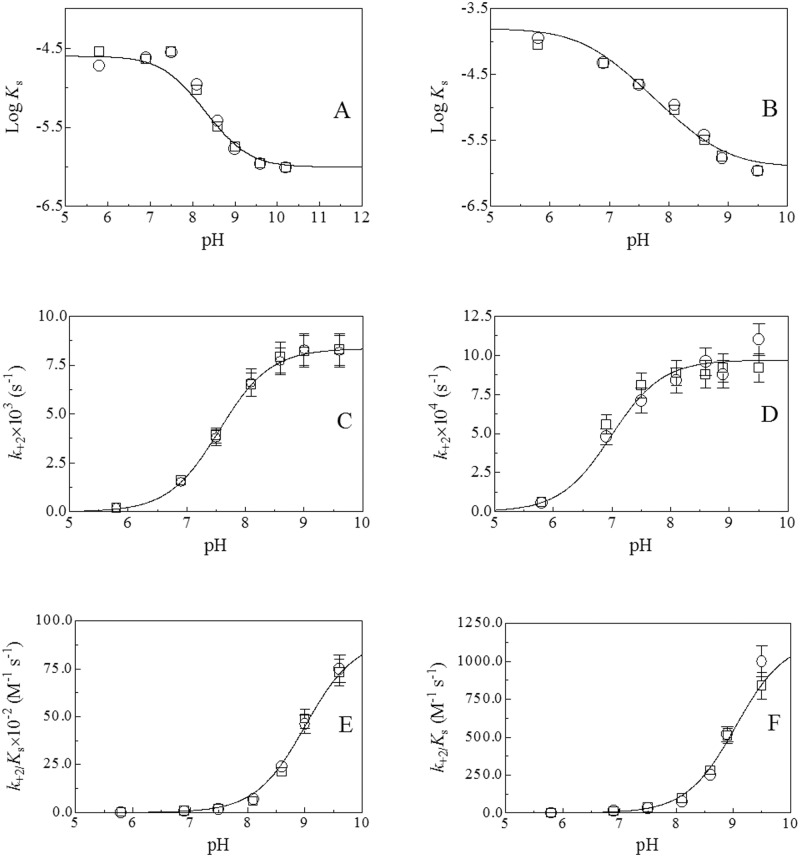
pH dependence of Log *K*_s_ (panels A and B), *k*_+2_ (panels C and D), and *k*_+2_/*K*_s_ (panels E and F) for HSA-mediated hydrolysis of NphOHe (panels A, C, and E) and NphODe (panels B, D, and F), at 22.0°C. Circles indicate data obtained under conditions where [HSA]≥5×[NphOHe] and [HSA]≥5×[NphODe]. Squares indicate data obtained under conditions where [NphOHe]≥5×[HSA] and [NphOHe]≥5×[HSA]. The continuous lines in panels A and B, C and D, and E and F were obtained from data analysis according to eqs (7)–(9), respectively, with values of parameters given in Table 2. Where not shown, the standard deviation is smaller than the symbol. For details, see text.

Values of catalytic parameters for the HSA-Tyr411-catalyzed hydrolysis of NphOHe and NphODe obtained between pH 5.8 and 9.5 (at 22.0°C) are summarized in S2 and S3 Tables. Fig. 3 shows the pH dependence of *K*_s_, *k*_+2_, and *k*_+2_/*K*_s_ values for the HSA-Tyr411-catalyzed hydrolysis of NphOHe and NphODe. Values of p*K*_a_ modulating the pH dependence of *k*_+2_/*K*_s_, *k*_+2_, and *K*_s_ were determined by data analysis according to eqs (7)–(9) [9,12,13,15–17]:

$$\text{Log}K_{\text{s}}=-\text{Log}K_{\text{s}}^{lim}+\text{Log}(\left(10^{-\text{pH}}+10^{-\text{p}K_{\text{unl}}}\right)/\left(10^{-\text{pH}}+10^{-\text{p}K_{\text{lig}}}\right))$$ (7)

$$k_{+2}=k_{+2}^{lim}/\left(1+(10^{-\text{pH}}/10^{-\text{p}K_{\text{lig}}})\right)$$ (8)

$$k_{+2}/K_{\text{s}}={\left(k_{+2}/K_{\text{s}}\right)}^{lim}/\left(1+(10^{-\text{pH}}/10^{-\text{p}K_{\text{unl}}})\right)$$ (9)

where *K*_s_^lim^, *k*_+2_^lim^, and (*k*_+2_/*K*_s_)^lim^ are the values corresponding to the alkaline asymptotes of *K*_s_, *k*_+2_, and *k*_+2_/*K*_s_.

**Table 2:** p*K*_unl_ and p*K*_lig_ values as well as of alkaline limiting values of *k*_+2_, *K*_s_, and *k*_+2_/*K*_s_ for the HSA-Tyr411-catalyzed hydrolysis of NphOAc; NphOHe, NphODe, and NphOMy, at 22.0°C.

NphOFA	*K*_s_	*K*_s_^lim^ (M)	*k*_+2_	*k*_+2_^lim^ (s^−1^)	*k*_+2_/*K*_s_	(*k*_+2_/*K*_s_)^lim^ (M^−1^ s^−1^)
NphOAc ^a^	p*K*_unl_ = 9.0±0.1	(6.6±0.7)×10^-5^			p*K*_unl_ = 9.0±0.1	(3.4±0.4)×10^4^
	p*K*_lig_ = 8.1±0.2		p*K*_lig_ = 8.1±0.2	2.1±0.2		
NphOHe ^b^	p*K*_unl_ = 8.9±0.2	(9.8±1.0)×10^-7^			p*K*_unl_ = 9.0±0.1	(9.1±1.0)×10^3^
	p*K*_lig_ = 7.5±0.2		p*K*_lig_ = 7.6±0.2	(8.3±0.8)×10^-3^		
NphODe ^b^	p*K*_unl_ = 8.9±0.1	(1.3±0.1)×10^-6^			p*K*_unl_ = 8.9±0.2	(9.6±1.1)×10^2^
	p*K*_lig_ = 6.9±0.2		p*K*_lig_ = 7.0±0.2	(9.8±1.1)×10^-4^		
NphOMy ^c^	p*K*_unl_ = 8.8±0.1	(1.4±0.1)×10^-6^			p*K*_unl_ = 8.9±0.1	(2.2±0.2)×10^2^
	p*K*_lig_ = 6.7±0.2		p*K*_lig_ = 6.9±0.2	(2.3±0.2)×10^-4^		

^a^ From [13].

^b^ Present study.

^c^ From [12].

According to linked functions [12,13,16–18], the pH dependence of *K*_s_ reflects the acidic p*K*_a_-shift of a single amino acid residue from free HSA (*i*.*e*., p*K*_unl_) to the HSA:NphOHe and HSA:NphODe complexes (*i*.*e*., p*K*_lig_). Moreover, the pH dependence of *k*_+2_ and *k*_+2_/*K*_s_ reflects the acid-base equilibrium of one apparent ionizing group in the HSA:NphOHe and HSA:NphODe complexes (*i*.*e*., p*K*_lig_) and in free HSA (*i*.*e*., p*K*_unl_), respectively. As expected [12,13,16–18], the p*K*_a_ value of free HSA (*i*.*e*., p*K*_unl_) is independent of the substrate whereas the p*K*_a_ values of the HSA:NphOHe and HSA:NphODe complexes (*i*.*e*., p*K*_lig_) depend on the substrate (Table 2).

### Competitive inhibition of the pseudo-esterase activity of HSA-Tyr411

**Fig 4:**
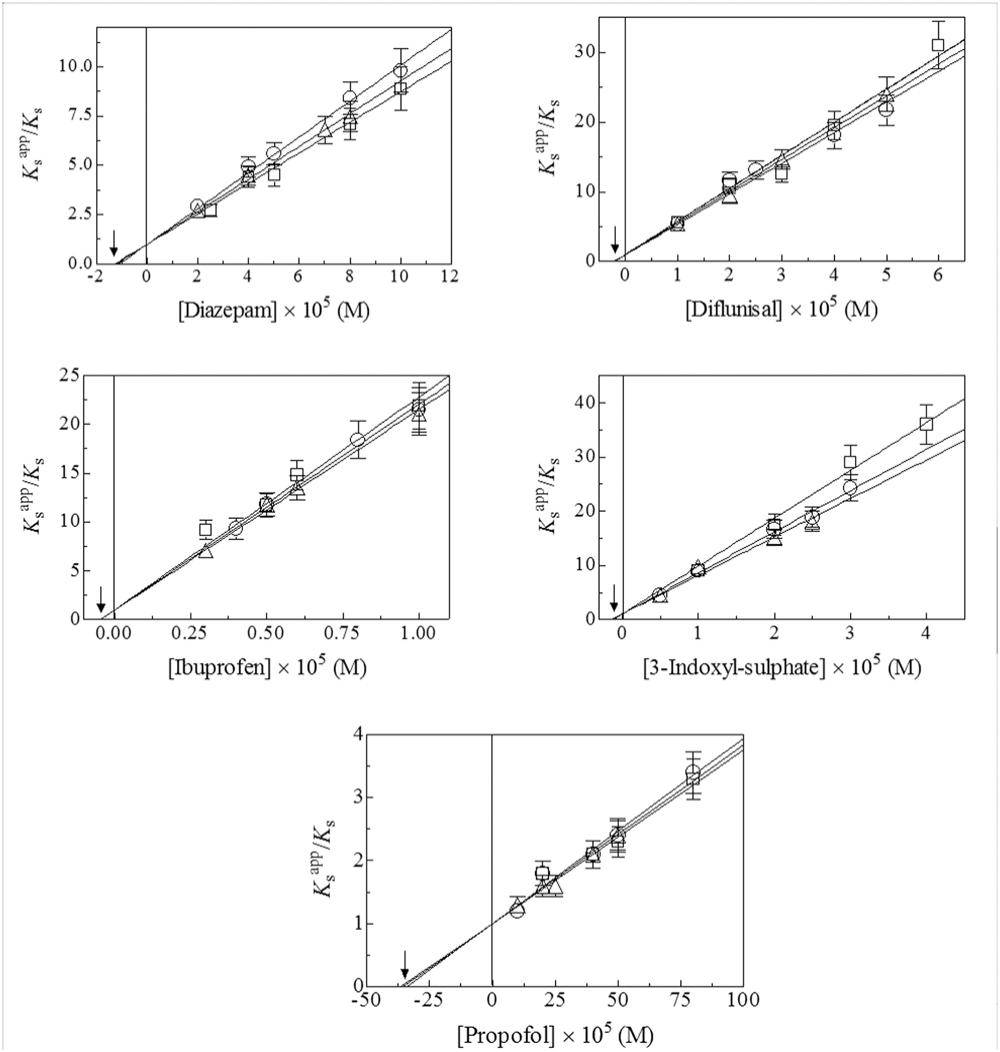
Competitive inhibitory effect of diazepam, diflunisal, ibuprofen, 3-indoxyl-sulfate, and propofol on the HSA-Tyr411-catalyzed hydrolysis of NphOHe (squares), NphODe (circles), and NphOMy (triangles). Data were obtained under conditions where [NphOHe] > 5×[HSA]. The analysis of data according to equation (10) allowed to determine values of *K*_I_ (indicated by arrows) reported in Fig. 5. Where not shown, the standard deviation is smaller than the symbol. For details, see text.

**Fig 5:**
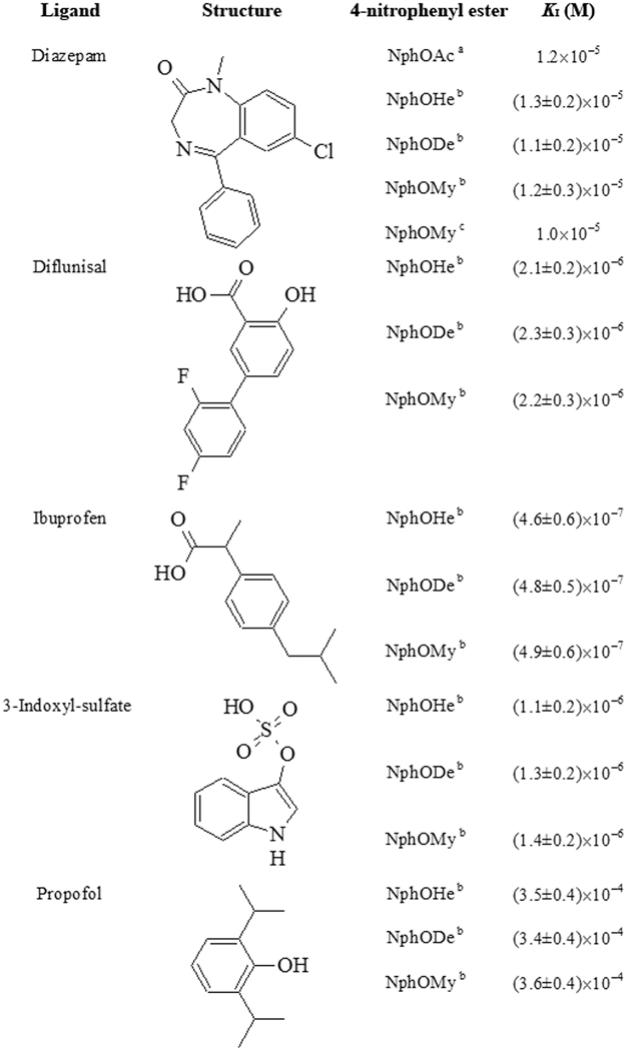
Values of *K*_I_ for the competitive inhibition of the HSA-Tyr411-catalyzed hydrolysis of NphOAc, NphOHe, NphODe, and NphOMy by diazepam, diflunisal, ibuprofen, 3-indoxyl-sulfate, and/or propofol, at pH 7.5 and 22.0°C. ^a^ From [13]. ^b^ Present study. ^c^ From [12].

As shown in Fig. 4, diazepam, diflunisal, ibuprofen, 3-indoxyl-sulfate, and propofol inhibit the Tyr411-catalyzed hydrolysis of NphOHe, NphODe, and NphOMy. As expected for the pure competitive inhibition mechanism [19], values of *K*_s_ for the HSA-Tyr411-catalyzed hydrolysis of NphOHe, NphODe, and NphOMy (Fig. 4) increase with the diazepam, diflunisal, ibuprofen, 3-indoxyl-sulfate, and propofol concentration whereas values of *k*_+2_ are unaffected by the ligand concentration (S1, S2, and S3 Figs.). The analysis of the linear dependence of the *K*_s_^app^/*K*_s_ ratio on the ligand concentration (*i*.*e*., [ligand]) according to equation (10) [19]:

$$K_{\text{s}}^{\text{app}}/K_{\text{s}}=(\left[\text{ligand}\right]/K_{\text{I}})+1$$ (10)

allowed to determine the values of the equilibrium dissociation constant for diazepam, diflunisal, ibuprofen, 3-indoxyl-sulfate, and propofol binding to the FA3-FA4 cleft of HSA (*i*.*e*., *K*_I_, corresponding to the absolute value of the *x* intercept of the linear plot). As expected for the pure competitive inhibition mechanism [19], values of *K*_I_ for diazepam, diflunisal, ibuprofen, 3-indoxyl-sulfate, and/or propofol binding to the FA3-FA4 cleft of HSA are independent of NphOAc [13], NphOHe (present study), NphODe (present study), and NphOMy (see [12] and present study) (Fig. 5). Values of *K*_I_ for diazepam, diflunisal, ibuprofen, 3-indoxyl-sulfate, and propofol binding to HSA here obtained (Fig. 5) agree with those reported previously [2,12,13,20–26].

## Discussion

The hydrolysis of NphOAc, NphOHe, NphODe, and NphOMy by HSA-Tyr411 (see [12–15] and present study) is reminiscent of that observed for acylating agents with proteases [27]. In fact, NphOAc [13], NphOHe (present study), NphODe (present study), and NphOMy [12] act as suicide substrates of HSA-Tyr411, values of the deacylation rate constant for all four substrates (*i*.*e*., *k*_+3_) being lower by several orders of magnitude than those of the acylation rate constant (*i*.*e*., *k*_+2_). Remarkably, HSA acylation appears to modulate ligand binding. In fact, HSA acylation by aspirin [28] increases the affinity of phenylbutazone and inhibits bilirubin and prostaglandin binding, thus accelerating the clearance of prostaglandins, which represents an additional mechanism of the aspirin anti-inflammatory action [29].

Kinetics for the hydrolysis of NphOHe and NphODe by HSA are pH dependent, reflecting the acidic p*K*_a_ shift of an apparently single ionizing group of HSA upon substrate binding. This could reflect the reduced solvent accessibility of the Tyr411 residue, representing the primary esterase site of HSA (see [9,12–14]), although long range effects could not be ruled out. The Tyr411 catalytic residue is located in the FA3-FA4 cleft, which is made by an apolar region forming the FA3 site and a polar patch contributing the FA4 site. The polar patch is centered on the Tyr411 side chain and includes Arg410, Lys414, and Ser489 residues [8,30,31]. The inspection of the three-dimensional structure of the ligand-free HSA [32] and of the molecular model of the HSA:4-nitrophenyl propionate complex [9] suggests that the observed pH effects (Fig. 3) could reflect the acidic p*K*_a_ shift of the Tyr411 residue upon substrate binding. This would render more stable the negative charge on the phenoxyl O atom of Tyr411, which appears to be hydrogen bonded to the carbonyl O atom of 4-nitrophenyl propionate [9], potentiating its nucleophilic role as an electron donor in the pseudo-esterase activity of HSA. Of note, the acidic shift of the p*K*_a_ value of the ionizing group affecting catalysis from 8.9±0.1 in ligand free-HSA to 8.1±0.2, 7.6±0.2, 7.0±0.2, and 6.8±0.2 in the HSA:NphOAc, HSA:NphOHe HSA:NphODe, and HSA:NphOMy complexes (see Table 2), respectively, depends on the length of the fatty acid tail. Therefore, it appears as the increase of the FA tail length brings about the progressive reduction of the water solvent accessibility, thus enhancing the hydrophobicity of the catalytic site and leading to a decreased p*K*_a_ of the ionizing group modulating the catalysis. Of note, the pH dependence of the Tyr411-associated esterase activity parallels the pH-dependent neutral-to-basic allosteric transition of HSA [3].

**Fig 6:**
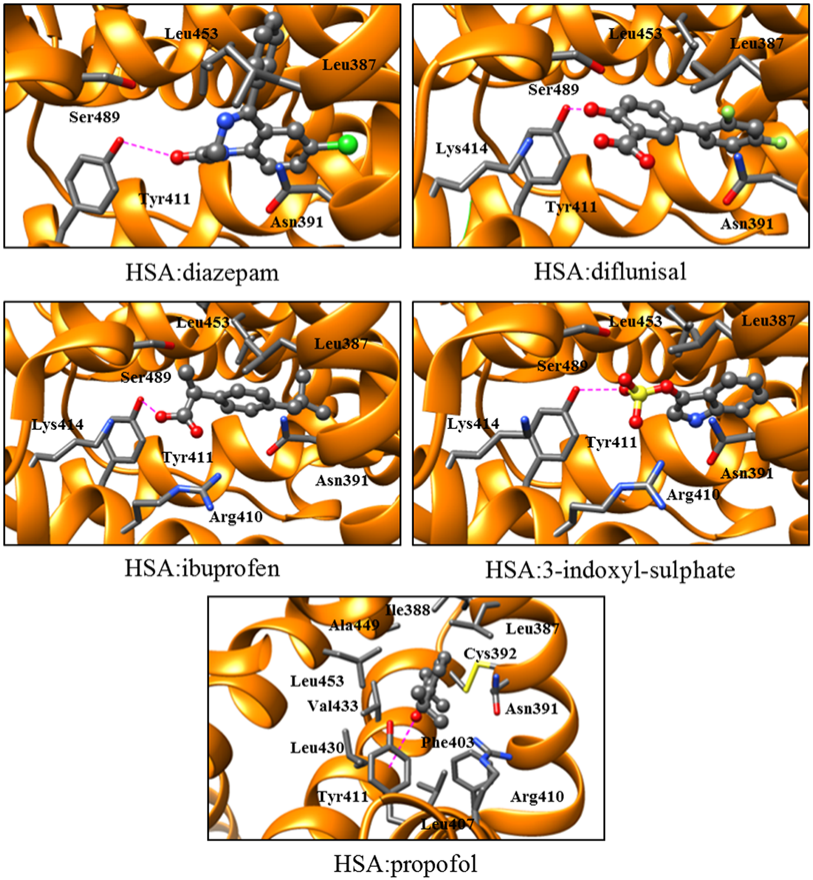
Three-dimensional structures of the HSA:diazepam, :diflunisal, :ibuprofen, :3-indoxyl-sulfate, and :propofol complexes. The ligands are shown in the ball-and-stick representation. The Tyr411 phenoxyl O atom of HSA is hydrogen bond to a O atom of diazepam, diflunisal, ibuprofen, and 3-indoxyl-sulfate. The Leu430 carbonyl O atom of HSA is hydrogen bond to the O atom of propofol. The hydrogen bonds are represented as red dashed lines. The PDB ID codes of HSA:diazepam, :diflunisal, :ibuprofen, :3-indoxyl-sulfate, and :propofol complexes are 2BXE, 2BXF, 2BXG, 2BXH, and 1E7A, respectively [8,30]. The pictures were drawn with the UCSF-Chimera package [46]. For details, see text.

Diazepam, diflunisal, ibuprofen, 3-indoxyl-sulfate, and/or propofol inhibit competitively the hydrolysis of NphOAc [13], NphOHe (present study), NphODe (present study), and NphOMy (see [12] and present study) (Fig. 5) by impairing the accessibility of 4-nitroplenyl esters to the Tyr411 catalytic center. In particular, diazepam, diflunisal, ibuprofen, and 3-indoxyl-sulfate bind to the center of the FA3-FA4 cleft, with one O atom being hydrogen bonded to the Tyr411 OH group (Fig. 6). On the other hand, propofol binds to the apolar region of the FA3-FA4 cleft with the phenolic OH group making a hydrogen bond with the carbonyl O atom of Leu430. Moreover, the aromatic ring of the propofol is sandwiched between the Asn391 and Leu453 side chains. Furthermore, one of the two isopropyl groups of propofol makes several apolar contacts at one end of the pocket, whereas the other is solvent exposed at the cleft entrance making close contacts with Asn391, Leu407, Arg410, and Tyr411 (Fig. 6) [8,30,31].

The different *K*_I_ values for diazepam, diflunisal, ibuprofen, 3-indoxyl-sulfate, and propofol binding to HSA (Fig. 5) agree with the selectivity of the FA3-FA4 cleft of HSA, which can be ascribed to the presence of a basic polar patch located at one end of the apolar FA3-FA4 cleft. Remarkably, diazepam, diflunisal, ibuprofen, and 3-indoxyl-sulfate are oriented with at least one O atom in the vicinity of the polar patch. On the other hand, the single polar hydroxyl group in the center of propofol does not interact with the polar patch of the FA3-FA4 cleft. Moreover, the FA3-FA4 cleft appears to adopt different ligand-dependent shapes, thus paying different free energy contributions for structural rearrangements [8].

## Conclusion

Due to the relevant physiological role of HSA in human plasma, *in vivo* implications can be argued from the present results. Accounting for the plasma levels of HSA (*i*.*e*., [HSA] = 7.5×10^-4^ M) [2] as well as of diflunisal, ibuprofen, and 3-indoxyl-sulfate plasma levels (*i*.*e*., [ligand]; see below) and *K*_I_ values for ligand binding (Fig. 4 and Fig. 5), the molar fraction (*i*.*e*., *Y*) of diazepam, diflunisal, ibuprofen, 3-indoxyl-sulfate, and propofol bound to plasmatic HSA was calculated according to equation (11) [33]:

$$[\text{HSA}]\times Y^{2}-([\text{ligand}]+[\text{HSA}]+K_{\text{I}})\times Y+[\text{ligand}]=0$$ (11)

The following therapeutic plasma levels of the drugs investigated are commonly reported at steady-state: (*i*) 0.3–0.4 μg/mL (*i*.*e*., 1.1×10^-6^ M to 1.1×10^-6^ M) of diazepam are recommended for anxiolytic effects, and *ca*. 0.6 μg/mL (*i*.*e*., 2.1×10^-6^ M) for control of seizures; higher concentrations might suggest misuse or abuse [34–36]; (*ii*) 90–110 μg/mL (*i*.*e*., 3.6×10^-4^ M to 5.3×10^-4^ M) of diflunisal are usually required for anti-inflammatory effects [37,38]; (*iii*) 20–40 μg/mL (*i*.*e*., 9.7×10^-5^ M to 1.9×10^-4^ M) are observed after a single oral anti-inflammatory dose of ibuprofen [39,40]; and (*iv*) 1–2 μg/mL (*i*.*e*., 5.6×10^-6^ M to 1.1×10^-5^ M) of propofol are necessary to maintain sleep [41,42]. Conversely, 3-indoxyl-sulfate is an uremic toxin accumulated in the plasma of chronic kidney disease patients and induces an oxidative stress in a variety of cells such as renal tubular cells, glomerular mesangial cells, vascular smooth muscle cells, vascular endothelial cells, and osteoblasts. Under pathological conditions, the plasma concentration of 3-indoxyl-sulfate ranges between 4 and 60 μg/mL (*i*.*e*., 1.9×10^-5^ M to 2.8×10^-4^ M) [43,44].

Since the plasma levels of diflunisal, ibuprofen, and 3-indoxyl-sulfate (see above) are higher than values of *K*_I_ for ligand binding to HSA by about 100 folds (see Fig. 4 and Fig. 5), the molar fraction of diflunisal, ibuprofen, and 3-indoxyl-sulfate bound to HSA is higher than 0.9, according to equation, (11). Although the commonly reported diazepam and propofol plasma levels (see above) are lower than the corresponding values of *K*_I_ for drug binding to HSA (see Fig. 4 and Fig. 5) by about 5 and 100 folds, respectively, the plasma HSA concentration (see above) is higher than *K*_I_ for by about 70 and 2 folds, respectively. According to equation (11), the molar fraction of diazepam and propofol bound to HSA in plasma is higher than 0.9 and 0.5, respectively.

As a whole, data here reported highlight the role of drugs diazepam, diflunisal, ibuprofen, and propofol as well as of the uremic toxin 3-indoxyl-sulfate to inhibit competitively the pseudo-esterase activity of HSA, Tyr411 representing the nucleophile. This aspect is appropriate since HSA acylation appears to modulate ligand binding [28,29] and the detoxification of several compounds [2,3]. Last, HSA not only acts as a carrier and as a detoxifier but also displays transient drug- and toxin-based properties, representing a case for “chronosteric effects” [45]. This opens the scenario toward the possibility of a drug- and toxin-dependent multiplicity of roles for HSA.

## Supporting Information

S1 Fig Effect of diazepam, diflunisal, ibuprofen, 3-indoxyl-sulphate, and propofol on the *k*_+2_ value for the HSA-Tyr411-catalyzed hydrolysis of NphOHe, at pH 7.5 and 22.0°C. The filled square on the ordinate indicates the *k*_+2_ value obtained in the absence of the ligands.
(DOC)

S2 Fig Effect of diazepam, diflunisal, ibuprofen, 3-indoxyl-sulphate, and propofol on the *k*_+2_ value for the HSA-Tyr411-catalyzed hydrolysis of NphODe, at pH 7.5 and 22.0°C. The filled square on the ordinate indicates the *k*_+2_ value obtained in the absence of the ligands.
(DOCX)

S3 Fig Effect of diazepam, diflunisal, ibuprofen, 3-indoxyl-sulphate, and propofol on the *k*_+2_ value for the HSA-Tyr411-catalyzed hydrolysis of NphOMy, at pH 7.5 and 22.0°C. The filled square on the ordinate indicates the *k*_+2_ value obtained in the absence of the ligands.
(DOCX)

S1 Table Values of α for the HSA-Tyr411-catalyzed hydrolysis of NphOHe and NphODE, at 22.0°C. (DOC)

S2 Table Values of catalytic parameters for the HSA-Tyr411-catalyzed hydrolysis of NphOHe, at 22.0°C. (DOCX)

S3 Table Values of catalytic parameters for the HSA-Tyr411-catalyzed hydrolysis of NphODe, at 22.0°C. (DOC)
