# Supplementary material for: Ligand Binding to the FA3-FA4 Cleft Inhibits the Esterase-Like Activity of Human Serum Albumin
Source: PLoS One. 2015 Mar 19;10(3):e0120603. doi: 10.1371/journal.pone.0120603 (PMC4366387; doi:10.1371/journal.pone.0120603)
Supplement: S2 Fig — The filled square on the ordinate indicates the k +2 value obtained in the absence of the ligands. (DOCX) [file pone.0120603.s002.docx]

**Figure S2.** Effect of diazepam, diflunisal, ibuprofen, 3-indoxyl-sulphate, and propofol on the *k*_+2_ value for the HSA-Tyr411-catalyzed hydrolysis of NphODe, at pH 7.5 and 22.0 °C. The filled square on the ordinate indicates the *k*_+2_ value obtained in the absence of the ligands.
