# Supplementary material for: Ligand Binding to the FA3-FA4 Cleft Inhibits the Esterase-Like Activity of Human Serum Albumin
Source: PLoS One. 2015 Mar 19;10(3):e0120603. doi: 10.1371/journal.pone.0120603 (PMC4366387; doi:10.1371/journal.pone.0120603)
Supplement: S1 Table — (DOC) [file pone.0120603.s004.doc]

**Table S1.** Values of α for the HSA-Tyr411-catalyzed hydrolysis of NphOHe and NphODE, at 22.0 °C.

------------------------------------------------------------------------------------------------------------------------

[NphOHe]≥5×[HSA] [NphOHe]≥5×[HSA]

------------------------------------- -------------------------------------

pH α pH α

-----------------------------------------------------------------------------------------

5.8 1.01 5.8 0.99

6.9 1.03 6.9 1.01

7.5 0.99 7.5 1.02

8.1 1.00 8.1 1.00

8.6 0.99 8.6 1.03

9.0 1.01 8.9 1.02

9.5 1.02 9.5 0.99

------------------------------------------------------------------------------------------------------------------------
