# Supplementary material for: Ligand Binding to the FA3-FA4 Cleft Inhibits the Esterase-Like Activity of Human Serum Albumin
Source: PLoS One. 2015 Mar 19;10(3):e0120603. doi: 10.1371/journal.pone.0120603 (PMC4366387; doi:10.1371/journal.pone.0120603)
Supplement: S2 Table — (DOCX) [file pone.0120603.s005.docx]

**Table S2.** Values of catalytic parameters for the HSA-Tyr411-catalyzed hydrolysis of NphOHe, at 22.0 °C.

----------------------------------------------------------------------------------------------------------------------------------------------------------------------------------

pH [HSA]≥5×[NphOHe] [NphOHe]≥5×[HSA]

----------------------------------------------------------------------- ----------------------------------------------------------------------

*K*_s_ *k*_+2_ *k*_+2_*/K*_s_ *K*_s_ *k*_+2_ *k*_+2_*/K*_s_

(M) (s^-1^) (M^-1^ s^-1^) (M) (s^-1^) (M^-1^ s^-1^)

----------------------------------------------------------------------------------------------------------------------------------------------------------------------------------

5.8 (2.9±0.3)×10^-5^ (2.0±0.2)×10^-4^ 6.9±0.7 (1.9±0.2)×10^-5^ (1.8±0.2)×10^-4^ 9.5±1.0

6.9 (2.3±0.2)×10^-5^ (1.5±0.2)×10^-3^ (6.4±0.7)×10^1^ (2.4±0.2)×10^-5^ (1.6±0.2)×10^-3^ (6.7±0.7)×10^1^

7.5 (2.9±0.3)×10^-5^ (2.8±0.3)×10^-3^ (9.7±1.3)×10^1^ (2.8±0.3)×10^-5^ (2.9±0.3)×10^-3^ (1.0±0.2)×10^2^

8.1 (9.4±0.8)×10^-6^ (6.6±0.7)×10^-3^ (7.0±0.7)×10^2^ (1.1±0.1)×10^-5^ (6.5±0.7)×10^-3^ (5.8±0.6)×10^2^

8.6 (3.2±0.3)×10^-6^ (7.7±0.7)×10^-3^ (2.4±0.2)×10^3^ (3.8±0.4)×10^-6^ (7.9±0.7)×10^-3^ (2.1±0.2)×10^3^

9.0 (1.8±0.2)×10^-6^ (8.3±0.8)×10^-3^ (4.6±0.5)×10^3^ (1.7±0.2)×10^-6^ (8.2±0.8)×10^-3^ (4.9±0.5)×10^3^

9.5 (1.1±0.1)×10^-6^ (8.2±0.8)×10^-3^ (7.5±0.7)×10^3^ (1.1±0.1)×10^-6^ (8.3±0.8)×10^-3^ (7.3±0.7)×10^3^

----------------------------------------------------------------------------------------------------------------------------------------------------------------------------------
