# Supplementary material for: Ligand Binding to the FA3-FA4 Cleft Inhibits the Esterase-Like Activity of Human Serum Albumin
Source: PLoS One. 2015 Mar 19;10(3):e0120603. doi: 10.1371/journal.pone.0120603 (PMC4366387; doi:10.1371/journal.pone.0120603)
Supplement: S3 Table — (DOC) [file pone.0120603.s006.doc]

**Table S3.** Values of catalytic parameters for the HSA-Tyr411-catalyzed hydrolysis of NphODe, at 22.0 °C.

----------------------------------------------------------------------------------------------------------------------------------------------------------------------------------

pH [HSA]≥5×[NphODe] [NphODe]≥5×[HSA]

----------------------------------------------------------------------- ----------------------------------------------------------------------

*K*_s_ *k*_+2_ *k*_+2_*/K*_s_ *K*_s_ *k*_+2_ *k*_+2_*/K*_s_

(M) (s^-1^) (M^-1^ s^-1^) (M) (s^-1^) (M^-1^ s^-1^)

----------------------------------------------------------------------------------------------------------------------------------------------------------------------------------

5.8 (8.9±0.9)×10^-5^ (6.0±0.6)×10^-5^ (6.8±0.7)×10^-1^ (1.1±0.1)×10^-4^ (5.6±0.6)×10^-5^ (5.1±0.6)×10^-1^

6.9 (4.6±0.2)×10^-5^ (5.6±0.6)×10^-4^ (1.2±0.1)×10^1^ (4.8±0.5)×10^-5^ (6.0±0.6)×10^-4^ (1.3±0.2)×10^1^

7.5 (2.3±0.2)×10^-5^ (8.1±0.8)×10^-4^ (3.5±0.5)×10^1^ (2.2±0.2)×10^-5^ (7.1±0.8)×10^-4^ (3.3±0.5)×10^1^

8.1 (9.4±0.8)×10^-6^ (8.9±0.8)×10^-4^ (9.5±1.0)×10^1^ (1.1±0.1)×10^-5^ (8.4±0.8)×10^-4^ (7.6±0.8)×10^1^

8.6 (3.2±0.3)×10^-6^ (8.8±0.9)×10^-4^ (2.8±0.3)×10^2^ (3.8±0.4)×10^-6^ (9.6±0.9)×10^-4^ (2.5±0.3)×10^2^

8.9 (1.8±0.2)×10^-6^ (9.2±0.9)×10^-4^  (5.1±0.5)×10^2^ (1.7±0.2)×10^-6^ (8.8±0.9)×10^-4^ (5.2±0.5)×10^2^

9.5 (1.1±0.1)×10^-6^ (9.2±0.9)×10^-4^ (8.4±0.8)×10^2^ (1.1±0.1)×10^-6^ (1.1±0.1)×10^-3^ (1.0±0.1)×10^3^

----------------------------------------------------------------------------------------------------------------------------------------------------------------------------------
